# Optimization of a therapeutic electromagnetic field (EMF) to retard breast cancer tumor growth and vascularity

**DOI:** 10.1186/s12935-014-0125-5

**Published:** 2014-12-07

**Authors:** Ivan L Cameron, Marko S Markov, W Elaine Hardman

**Affiliations:** Department of Cellular and Structural Biology, University of Texas Health Science at San Antonio, 7703 Floyd Curl Drive, San Antonio, Texas 78229 USA; Research International, Williamsville, New York 14221 USA; Department of Biochemistry and Microbiology, Joan C. Edwards School of Medicine, Marshall University, 1600 Medical Center Dr., Huntington, 25755 West Virginia

**Keywords:** Electromagnetic field (EMF), Breast cancer, Angiogenesis, Immunohistochemical, Endothelial cell marker (CD31)

## Abstract

**Background:**

This study provided additional data on the effects of a therapeutic electromagnetic field (EMF) device on growth and vascularization of murine 16/C mammary adenocarcinoma cells implanted in C3H/HeJ mice.

**Methods:**

The therapeutic EMF device generated a defined 120 Hz semi sine wave pulse signal of variable intensity. Murine 16/C mammary adenocarcinoma tumor fragments were implanted subcutaneously between the scapulae of syngeneic C3H mice. Once the tumor grew to 100 mm^3^, daily EMF treatments were started by placing the cage of mice within the EMF field. Treatment ranged from 10 to 20 milli-Tesla (mT) and was given for 3 to 80 minutes either once or twice a day for 12 days. Tumors were measured and volumes calculated each 3–4 days.

**Results:**

Therapeutic EMF treatment significantly suppressed tumor growth in all 7 EMF treated groups. Exposure to 20mT for 10 minutes twice a day was the most effective tumor growth suppressor. The effect of EMF treatment on extent of tumor vascularization, necrosis and viable area was determined after euthanasia. The EMF reduced the vascular (CD31 immunohistochemically positive) volume fraction and increased the necrotic volume of the tumor. Treatment with 15 mT for 10 min**/**d gave the maximum anti-angiogenic effect. Lack of a significant correlation between tumor CD 31 positive area and tumor growth rate indicates a mechanism for suppression of tumor growth in addition to suppression of tumor vascularization.

**Conclusion:**

It is proposed that EMF therapy aimed at suppression of tumor growth and vascularization may prove a safe alternative for patients whether they are or are not candidates for conventional cancer therapy.

## Background

A novel electromagnetic field has been reported to safely reduce growth and vascularization of an implanted breast cancer in mice [1,2]. The purpose of the present study was to perform additional animal studies using the “Complex Magnetic Field Generating Device” (referred to as “magnetic device”) on tumor growth in a mouse tumor model. In this study, the effects of varying treatment doses (magnetic field intensities) were evaluated using the murine 16/C mammary tumor in syngeneic C3H mice.

**Figure 1:**
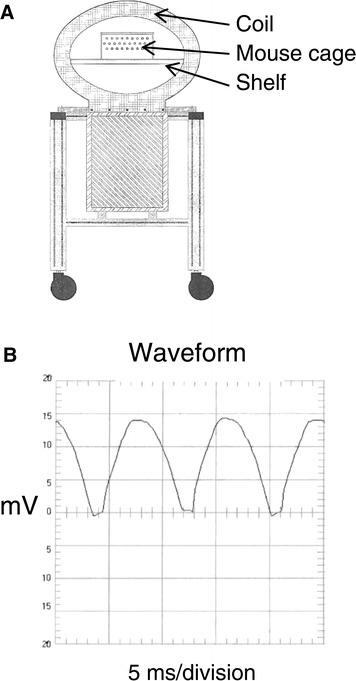
**The complex magnetic field device and illustration of field generated. A**. Drawing illustrating the “Complex Magnetic Field Device” used in the current study. The ellipsoidal coil with 14” by 21” diameters targets the EMF exposure area. The magnetic flux density, as measured within the perforated Plexiglass wall of the exposure chamber (mouse cage), produces a homogeneous signal of 10, 15 or 20 mT. The temperature change within the chamber during mice exposures to therapeutic EMF did not exceed 1˚C. **B**. The device generates a pulsating half (semi) sine wave magnetic field with a frequency of 120 pulses per second, as shown. A small DC component occurs between two semi-sine waves and a slight distortion is observed at the front of part of the semi-sine wave. The type of signal flip-flops the negative part of the sine wave into a positive thus creating a pulsed semi-sine wave.

The magnetic device used was supplied and calibrated by Bio-Dynamics. This magnetic device generated a rectified semi-sine wave signal of 10 to 20 milli-Tesla (mT) magnetic field intensity at 120 pulses per second. Diagrams of the device and of the waveform are shown in Figure 1. The cage of tumor-bearing mice was placed on the shelf inside the coil. Mice were treated with varying treatment intensities and durations to determine if the therapeutic EMF caused a significant decrease in the tumor growth. Table 1 lists the different therapeutic EMF conditions included in the current report. Data groups 1, 3, 6, 7 are from [2] and new unpublished data has been added for groups 2, 4, 5, 8.

**Table 1:** **EMF exposure conditions used in this study**

**Group number**	**EMF exposure condition**
1.	Control (0 mT) sham exposure
2.	10 mT 3 minutes per day
3.	10 mT 10 minutes per day
4.	10 mT 40 minutes per day
5.	10 mT 40 minutes twice per day
6.	15 mT 10 minutes per day
7.	20 mT 10 minutes per day
8.	20 mT 10 minutes twice per day

There were 20 mice in group 1, all other groups had 10 mice.

Adding the new unpublished experimental data allowed a more complete analyses of EMF time and field intensity needed to best retard tumor growth. The additional new data also allowed further testing of the hypothesis that the therapeutic EMF reduces tumor growth rate by reduction in the extent of tumor vascularization (anti-angiogenesis) leading to an increase in the extent of tumor necrosis [2]. This report has two separate result sections. The first section deals with effects of EMF therapy on tumor growth and the second section deals with the effects of the EMF on the vascular, necrotic and viable volume density fractions of the tumors. In brief, the results of this study demonstrate that: 1) tumor growth retardation was related to increased magnetic field intensity but not to increased time at a constant intensity and 2) the magnetic field exposure significantly inhibited tumor vascularization.

## Results and discussion

### Section 1: Effect of therapeutic electromagnetic field intensity (the treatment) on growth of subcutaneously implanted C/16 murine mammary adenocarcinomas (the tumor) in mice

**Figure 2:**
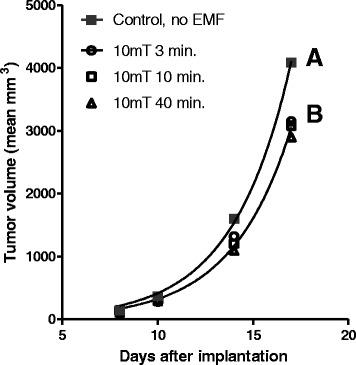
**Mean tumor volumes of control and treated mice, constant intensity for increasing time.** Mean tumor volume of control mice (0 EMF, regression line **A**) or groups of mice exposed to 10mT EMF for increasing time each day starting on day 8 after tumor cell implantation (regression line **B**). Statistical analyses (non-linear analyses of exponential growth curves, Prism™) showed that the mean tumor volume of all EMF treated groups on day 17 was significantly smaller than the untreated (sham) control groups. However, there was not a significant difference between treatment groups.

**Figure 3:**
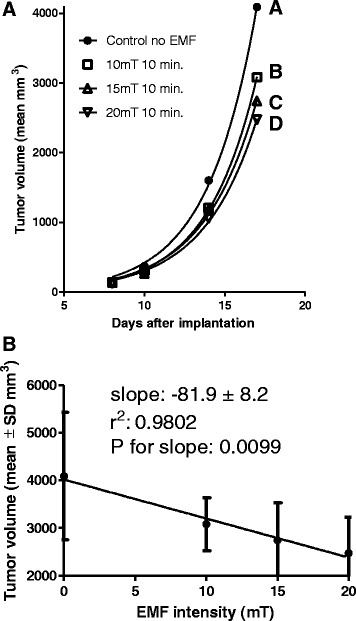
**Mean tumor volumes of control and treated mice, increasing intensity for constant time. A**. Mean volume (mm^3^) of the murine 16/C breast adenocarcinoma tumor in control group and in the groups of mice exposed to 10, 15 or 20 mT EMF for 10 minutes once a day. Tumors were measured on day 8 just before the first exposure and again on days 10, 14, 17 and 20. ANOVA followed by SNK statistical analysis of group data (Table 2) revealed that by day 17 the tumors in the control group were significantly larger than the tumors of all treatment groups. **B**. Linear regression analyses of tumor volume at 17 days after implantation of mice receiving either 0, 10 15 or 20 mT EMF for 10 minutes per day illustrating the significant linear decrease in tumor volume with increasing EMF intensity.

Therapeutic EMF treatment was for 12 days starting on day 8 after tumor implantation. By day 8, all tumors had grown to measurable size (about 5 mm in diameter). Tumor volume data collection was then initiated. Table 1 shows the treatment conditions of each group. The treatment groups were subdivided to determine the effect of: 1) increasing the time of the treatment at a constant intensity (Figure 2) or 2) increasing the intensity of treatment for a constant time (Figure 3). The data in Figure 2 and Table 2 indicate that EMF exposure at 10mT intensity did significantly suppress tumor growth compared to control but that increasing the length of exposure (from 3 minutes to 10 or 40 minutes per day) at 10mT intensity did not result in additional suppression of tumor mass. In fact, there was no difference in the non-linear regressions (that is, the null hypothesis that the exponential growth equations were the same was accepted, analyses by Prism™) of tumor growth at the three times thus Prism™ provided a single line (labeled B in Figure 2). The data in Figure 3A shows increasing intensity of EMF treatment from 10 to 15 to 20 mT did result in dose responsive suppression of tumor growth compared to control (analyses of the exponential growth equation resulted in rejecting the null hypothesis that the lines were the same, Prism™). By day 14, the tumor volume of groups 7 and 8 (the two 20mT groups) was significantly less (p = 0.01) and by day 17, the tumor volume of all groups was significantly less than of the control group, p = 0.001 (2 way ANOVA, Prism™). Figure 3B shows that increasing the intensity of EMF treatment from 10 to 15 to 20 mT at 10 minutes per treatment did significantly correlate with decreased tumor volume. This slowing of tumor growth is also supported by the increase in the calculated tumor doubling times from 2.12 days (control) to 2.16 days (at 10 mT) to 2.29 days (at 15 mT) to 2.304 days (at 20 mT) (non-linear analyses of exponential growth curves, Prism™).

**Table 2:** **Tumor volumes for each group, as defined in Table** 1 **at 8, 10, 14 and 17 days after implantation of tumors**

**Group**	**Day 8**	**Day 10**	**Day 14**	**Day 17**
1	138.5 ± 37.2	370.3 ± 184.0	1602.0 ± 845.2	4089.1 ± 1341.6^a^
2	130.9 ± 39.2	282.3 ± 140.9	1320.8 ± 529.0	3149.8 ± 727.0
3	137.1 ± 37.0	308.0 ± 125.8	1207.2 ± 445.5	3081.9 ± 553.0
4	111.2 ± 36.6	294.9 ± 132.2	1095.2 ± 382.6	2901.9 ± 696.2
5	129.8 ± 39.2	275.4 ± 114.1	1306.7 ± 440.3	3194.5 ± 524.5
6	132.6 ± 43.3	264.9 ± 96.2	1181.5 ± 630.9	2742.2 ± 784.6
7	123.4 ± 44.0	261.1 ± 166.3	1057.5 ± 528.6	2473.0 ± 756.5
8	142.2 ± 44.3	225.9 ± 81.8	922.0 ± 303.7	2402.8 ± 562.6
P value^b^	0.71	0.24	0.05	0.0001

^a^Means of the tumor volume for each group were not significantly different until day 14. ANOVA followed by SNK showed that on Day 14 the tumor volume of group 1 was significantly greater than that of groups 7 and 8 and that on Day 17, the tumor volume of Group 1 was significantly greater than that of all other groups. Group 1 had 20 mice, all other groups had 10 mice.

^b^P value from the ANOVA. The decreasing p value with time indicates that the differences between groups were increasing with time.

Tumor volume (mm^3^, mean ± SD).

Thus, a brief EMF exposure once or twice a day for nine days resulted in a significant decrease in the mean tumor size compared to the control group. The specific claim supported by the analysis of the tumor growth data is that all treated groups demonstrate slower tumor growth than in the control (non-EMF treated) group. Exposure to 10 or 15 or 20mT for 10 minutes per day caused a dose dependent suppression of the mean tumor volume to values 25% to 41% of the mean tumor volume of the control group.

At 20 days after tumor implantation, the non-EMF treated group of mice had 65% survival. Survival of mice in the EMF treated groups was dose dependently increased to 93% in the groups that received the 4 highest EMF dose levels (p = 0.017 for difference from non-EMF controls by Fishers Exact test, SAS.)

Microscopic pathological findings indicated no evidence of toxicological or biological abnormalities in any of the mouse tissues except the spleen. Some of the mice in each of the eight groups had extramedullary hematopoiesis. The incidence of extramedullary hematopoiesis in control mice was 4/10 while the incidence in EMF treated mice ranged from 3/10 in group 3 to 8/10 in group 8 with no apparent relationship to EMF treatment. The increased extramedullary hematopoiesis was probably due to non-specific stimulation of the immune system by the presence of the tumor. The implanted tumors all showed pathology typical of mammary gland neoplasms.

### Section 2: Effect of an electromagnetic field (the treatment) on vascular area and viable and necrotic tumor volume of subcutaneously implanted C/16 murine mammary adenocarcinomas (the tumor) in mice

Tissue cells receive oxygen and nutrients by diffusion from blood vessels. However, cells must be located within about 150 μm of a blood vessel for diffusion to adequately meet the oxygen and nutrient requirements for cell viability. Thus, growth and viability of the tumor is dependent on vascularization of the tumor by angiogenesis (the formation of new blood vessels). If the production of tumor cells is faster than vascularization of the new tumor tissue, then those cells further than 75 to 150 μm from a blood vessel will die. Any intervention that interferes with tumor angiogenesis can limit the growth of a tumor. This second section of the report deals with the effects of the various EMF treatment protocols on the extent of tumor vascularization and on the extent of viable and necrotic tumor volume fraction in tumors excised from mice at the termination of the experiment, 20 days after tumor transplantation and following 12 days of treatment. Data on tumors from mice that died prior to the termination of the experiment, at 20 days, were not included in this section of the report.

### Immunohistochemistry and morphometric analysis of cryosections

**Figure 4:**
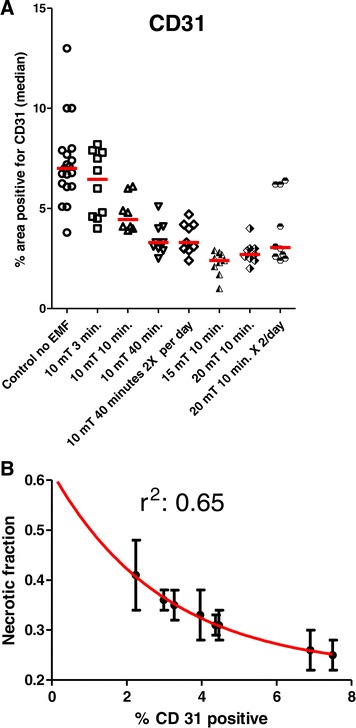
**Vascular and necrotic tumor fractions after EMF treatment. A**. Therapeutic EMF on the percent of tumor area immunohistochemically positive for CD 31 (a measure of tumor vascular volume fraction). The mean CD 31- positive area of each tumor treatment group is shown in the scattergram. Statistical analysis (ANOVA followed by SNK post-hoc test) revealed the mean of CD 31 positive area of control group # 1 to be significantly greater than of treatment groups 3 to 8 and groups 3 and 4 to be significantly greater than group 6. **B**. Significant, one phase decay relationship between CD 31positive area and the necrotic fraction of tumors after EMF treatment.

The results for the CD 31 positive area expressed as the percent of area of the total tumor area, are summarized in Table 3 and Figure 4. Figure 4A illustrates that the percent of CD 31 positive area was decreased by EMF treatment. Figure 4B shows the significant negative relationship between the necrotic fraction and the percent of CD 31 area*.* The EMF treatment at 15 mT per day gave the largest extent of necrotic tumor volume (least fraction of % area positive for CD 31) and approached the plateau of maximum therapeutic effectiveness in this study.

**Table 3:** **Effect of the EMF treatments on the extent of vascular (CD31 positive) necrotic and viable tissue in tumors taken from mice sacrificed 20 days after tumor implantation**

**Group**	**N**	**Total tumor volume (mm** ^**3**^ **)**	**Viable tumor volume (mm** ^**3**^ **)**	**Necrotic tumor volume (mm** ^**3**^ **)**	**Necrotic fraction**	**% CD 31 positive**
1	11	5084.7 ± 1257	3776.0 ± 884.4	1308.7 ± 425.9	0.25 ± 0.03	7.50 ± 2.4
2	8	4442.3 ± 884.6	3282.2 ± 691.1	1160.1 ± 268.6	0.26 ± 0.04	6.90 ± 2.0
3	6	4077.5 ± 579.8	2796.4 ± 392.1	1281.1 ± 225.0	0.31 ± 0.03	4.46 ± 1.1
4	9	3598.9 ± 964.2	2475.0 ± 691.7	1123.9 ± 278.3	0.31 ± 0.02	4.37 ± 0.7
5	9	4330.8 ± 295.4	2818.1 ± 614.4	1512.7 ± 304.6	0.35 ± 0.03	3.27 ± 0.8
6	7	3799.0 ± 480.4	2221.8 ± 753.9	1577.2 ± 663.2	0.41 ± 0.07	2.24 ± 1.1
7	8	3958.8 ± 244.6	2534.7 ± 497.8	1424.1 ± 255.7	0.36 ± 0.02	2.99 ± 0.6
8	9	3678.1 ± 599.3	2450.8 ± 397.6	1227.3 ± 290.9	0.33 ± 0.05	3.96 ± 1.5

Analysis of variance followed by a Student-Newman-Keuls multiple range test showed the following significant difference (p < 0.05) between the group means:

Volume of viable tumor – the viable tumor volume of group 1 was significantly greater than the viable tumor volume of groups 3 to 8; the viable tumor volume of group 2 was significantly greater than the viable tumor volume of group 6.

Necrotic fraction – the necrotic fractions of groups 1 and 2 were significantly less than the necrotic fractions of groups 3 and 8; the necrotic fraction of group 6 was significantly greater than the necrotic fraction of all other groups.

% CD 31 positive – the CD 31 positive areas (volume fraction) of groups 1 and 2 were significantly greater than the CD 31 positive area of groups 3 to 8; the CD 31 positive areas of groups 3 and 4 were significantly greater than the CD 31 positive area of group 6.

Total tumor volume – the total tumor volume of group 1 was significantly greater than the total tumor mass of all other groups.

All values are mean ± SD.

**Figure 5:**
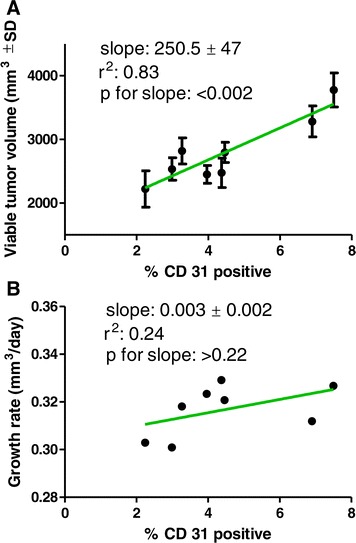
**Relationships between CD32 positive fraction, viable tumor volume and growth rates of EMF treated tumors. A**. The significant, linear relationship between the CD 31 positive fraction and the viable tumor volume of tumors. **B**. The non-significant relationship between the CD 31 positive fraction and the growth rate of the tumors. Taken together, these data illustrate that blood supply (CD 31 positive fraction) is critical for tumor viability but is not related to tumor growth.

The results illustrated in Figure 5A show that the % of CD 31 positive area was significantly related to the viable tumor fraction. However, Figure 5B illustrates that the CD 31 positive area is not significantly related to tumor growth. This could be interrupted to mean that blood supply is critical to tumor survival however adequate blood supply does not guarantee tumor growth. Cells may be dying or cell cycle may be arrested, thus growth will be slowed, even in the presence of adequate blood supply.

### Review of EMF treatment conditions used to retard tumor growth

The EMF exposure conditions used in the present and past literature reports are summarized in Table 4. The data in Table 4 indicates significant reduction of tumor growth in the different cancer tissue cell types studied. The effective pulse frequencies used range from 0.16 to 480 Hz and from 0.6 mT to 250 mT. All treatments were given either once or twice a day. Both sine wave and semi sine wave frequency signals significantly retarded tumor growth. No known comparative study has been reported to determine the wave form that best retards tumor growth [3].

**Table 4:** **A sample of literature reports on effects of electromagnetic field types on growth of cancerous tumors in animal hosts** ^**a**^

**Type of tumor**	**Frequency Hz and (pps)**	**Intensity Tesla**	**Exposure min (m), (hr), or sec/day (d)**	**Significant growth retardation yes or no**	**References**
Melanoma	25	2-5 mT	3 hr/d	Yes	Hu et al. 2010 [4]
Hepatoma	100	0.7 mT	1 hr 3x/d	Yes	Wen et al. 2011 [5]
Colon	50	2.5 & 5.5 mT	70 m/d	Yes	Tofani et al. 2002 [6]
Mammary	12 & 460	9 mT	10 m on alternate days	Yes	Bellossi & Desplasi 1991 [7]
MX-1	50	15-20 mT	3 hr/d	Yes	Berg et al. 2010 [8]
Carcinogen induced	0.8	100 mT	8 hr/d	Yes	Seze et al. 2000 [9]
Mammary	120^b^	10-20 mT	10 m/d	Yes	Williams 2001 [2]
Mammary	120^b^	10 & 20 mT	3 to 80 m/d	Yes	Cameron et al. this report
Mammary	120^b^	15 mT	10 m/d	Yes	Cameron et al. 2005 [1]
Mammary	1	100 mT	60 to 180 m/d	No	Tataova et al. 2011 [10]
	1		360 m/d	Yes	
Sarcoma	0.16 – 1.3	0.6 – 2.0 T	15 m/d	Yes	Zhang et al. 2002 [11]
Melanoma	50	5.5 mT	70 m/d	Yes	Tofani et al. 2003 [12]
Sarcoma	50	250 mT	80 sec/d	Yes	Yamaguch et al. 2006 [13]

^a^This sample is not comprehensive but is judged to be a reasonably representative.

^b^A semi sine wave was used in these studies, other studies used a sine wave signal.

The current study found that lengthening the duration of exposure to a 10 mT, 120 Hz semi sine wave EMF signal from between 3 to 40 minutes once a day did not significantly increase retardation of tumor growth. However, increasing the intensity of exposure from 10 to 15 to 20 mT for 10 minutes did dose responsively increase the tumor doubling time. Exposure to 20 mT for 10 minutes twice a day gave the longest lengthening of tumor growth doubling time. In another study Tatatova et al. [10] reported that increasing daily exposure to 1 Hz at 100 mT for 60 or 180 min did not cause significant retardation of tumor growth but exposure for 360 minutes twice a day did cause a significant reduction.

None of the EMF conditions reviewed resulted in tumor regression. Thus more research is still needed on the optimization of EMF exposure condition for cancer therapy however slowed tumor growth without adverse side effects could still provide clinical benefit for patients in combination or independent from conventional cancer chemo- or irradiation therapies.

### Mechanism of action of EMF on retardation of tumor growth

Data in the current and past reports [1,2] suggests that a therapeutic EMF semi-sine wave at 120 Hz at 10 to 20 mT for 10 minutes a day caused a significant retardation of tumor growth accompanied by a decrease in tumor vascularization and an increase in tumor necrotic volume fraction. Figure 5 reveals the vascular volume fraction (CD 31 positive) of the tumor to be an excellent predictor of the necrotic tumor volume fraction and Figure 4 shows that an EMF intensity of 15 mT for 10 minutes a day was an optimal exposure, in this study, to decrease tumor vascular area and to increase the resulting tumor cell necrosis.

**Figure 6:**
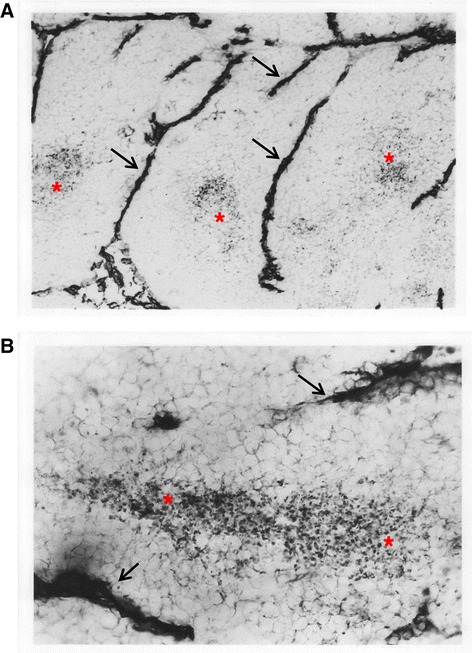
**Photomicrographs of CD31 immunostained tumor. A**. 100X original magnification. **B**. 250X original magnification. The photomicrographs are of 12 to 15 μm thick sections of a C/16 murine mammary adenocarcinoma after immunohistochemistry to detect the presence of CD 31 antigen, a marker for blood vessels. Blood vessels (arrowheads) are visualized by the presence of the dark stain for CD 31. Areas of viable tissue are seen adjacent to the blood vessels. Necrotic areas (*) are located further from the blood vessels and contain condensed nuclei and cell fragments. Notice the regular spacing of necrotic and viable areas.

**Figure 7:**
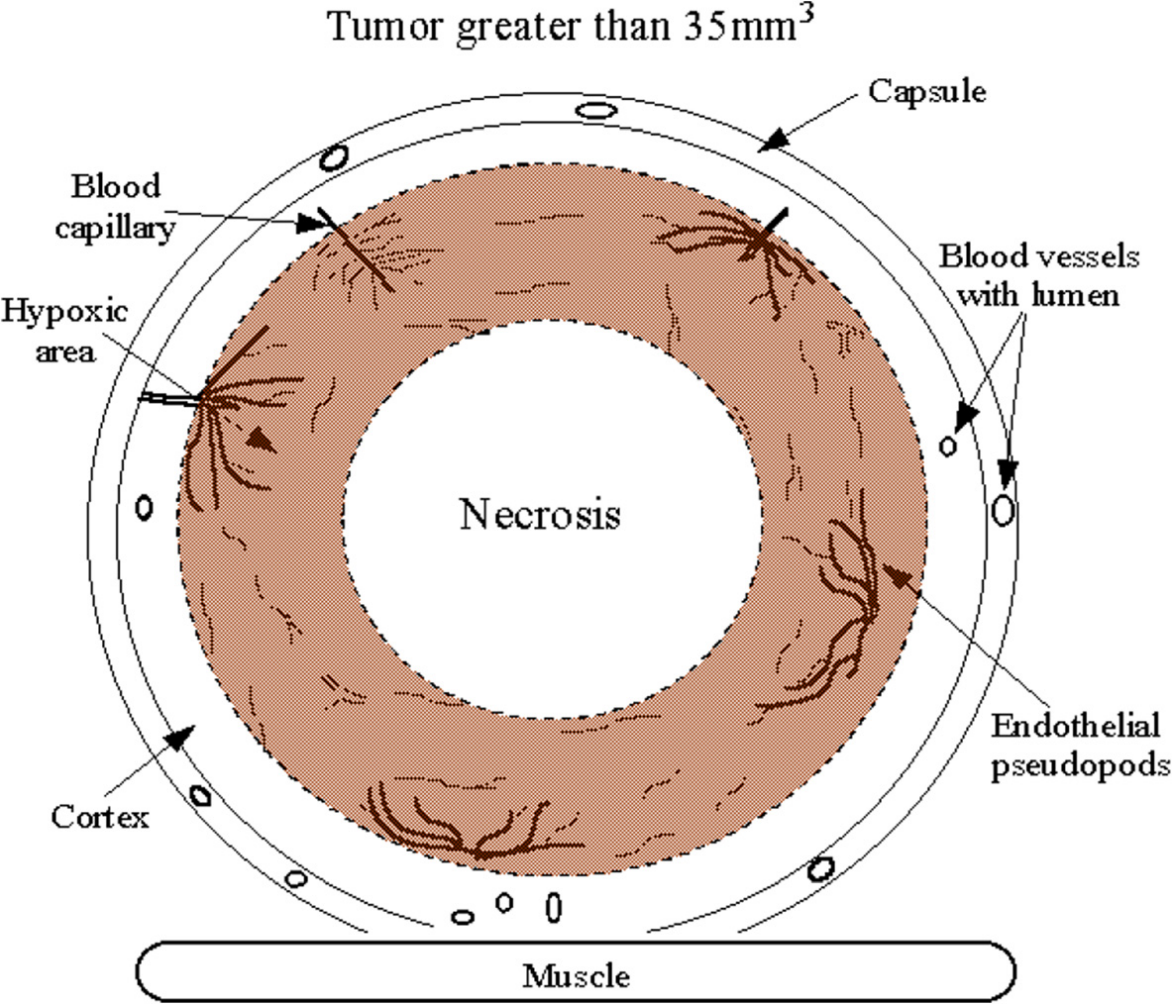
**Cross section of a human breast cancer tumor.** This drawing (from Cameron et al. [5]) depicts a cross section of a human breast cancer tumor. The tumor has a well vascularized capsule. An outer cortex underlies the capsule for 100 to 150 μm. Immunohistochemistry shows that a hypoxia inducing factor HIF-α rich subcortical region is present at distances greater than 100 to 150 μm from blood vessels. The HIF producing region is thought to produce vascular endothelial growth factors causing an observed sprouting of endothelial cell pseudopods and neo-angiogenesis. Cells further away from this neo-angiogenesis zone lack essential nutrients and oxygen and become necrotic. Thus this angiogenesis process is proposed to be hypoxia driven and is also proposed to be a main target of therapeutic EMF therapy.

Figures 6, A,B and 7 (from Cameron et al. [14]) illustrate that tumor cancer cells do not survive at distances of greater than 100–150 μm from a blood vessel. Tumor cancer cells that lack adequate oxygen become hypoxic and begin to produce hypoxia inducing factor (HIF- α). Vascular endothelial cells respond by sprouting endothelial cell pseudopods to project into the hypoxic regions of tumor cells and then form a vacuole/lumen that allows entrance of blood element, nutrients and oxygen into the hypoxic cell area [14]. The surviving tumor cells can then continue cell growth and proliferation. This vascularization process is termed tumor angiogenesis and any condition that interferes with this process is said to be anti-angiogenic. The current report and a prior report [14] demonstrate that an EMF treatment of 15 mT for 10 minutes a day is a safe and effective therapy for retardation of tumor vascularization, tumor growth and metastasis.

### Therapeutic EMF treatment options

Neither the current study nor any of the past studies on use of EMF on tumor growth (Table 4) give evidence that EMF treatment resulted in tumor regression to smaller size. From this observation one can conclude that use of therapeutic EMF does not appear to provide a possible sole (stand-alone) cure for tumor cancer patients. However, therapeutic EMF has proved a safe and effective adjuvant therapy when used in conjunction with gamma irradiation treatment [1]. The proposed reason for combining both therapeutic EMF and irradiation therapies was that each treatment focused on a different target. Irradiation and most cancer chemotherapy drugs focus on direct killing of rapidly proliferating cells while EMF appears to focus on retarding of cancerous tumor angiogenesis. The only other place in the unwounded adult human body that might require significant angiogenesis is during regrowth of uterine mucosal vascularization following menstruation in women. Thus harmful side effects of therapeutic EMF on angiogenesis in the normal adult unwounded body are very limited. The anti-angiogenesis action of therapeutic EMF appears to deprive the cancer cells of needed nutrients and oxygen and they die. Anti-angiogenesis agents can also interfere with tumor vascular metastasis [1].

Thus one can reasonably expect an additive or perhaps a synergistic effect of combining two different targets (anti-angiogenesis and anti-rapidly dividing cells) for treatment of cancer. Given that rapidly dividing cell populations are normally present throughout the body it follows that vascular delivery of cancer chemotherapeutic drugs that target dividing cells can also cause a number of harmful side effects in rapidly dividing normal tissues. However, irradiation therapy can be targeted to cancerous areas to help minimize this harmful side effect. In one study the combination of gamma irradiation and therapeutic EMF did have a significant additive inhibitory effect on tumorous breast cancer growth and metastasis [1].

## Conclusions

It is concluded that an appropriate therapeutic EMF as summarized in this report can safely reduce tumor growth and vascularization and can be a useful adjuvant for increasing the efficacy of conventional cancer therapies [1,8,12,15,16].

## Materials and methods

The C3H/HeJ mice used were obtained from the Frederick Cancer Research and Development Center of the National Cancer Institute (Frederick, MD). The C3H mice were approximately 7 weeks of age at the initiation of the study. Mice were housed in plastic micro-isolator cages with sterile hardwood bedding. They received standard laboratory diet and filtered tap water *ad libitum.* Air temperature and relative humidity in the animal rooms were controlled at 74 ± 2 F and approximately 50 ± 10%, respectively. Lights in the animal rooms were operated automatically on 12-hour light/dark cycles. The murine 16/C mammary adenocarcinoma tumor was used. Tumors were maintained in routine subcutaneous (s.c.) passage *in vivo* in C3H mice prior to implantation of tumor fragments.

Tumor implantation: Equal sized fragments of a C/16 murine mammary adenocarcinoma were implanted subcutaneously between the scapulae of C3H mice. EMF treatment was started on day 8 after tumor implantation and continued for 12 days. By day 8, all tumors had grown to measurable size (about 5 mm) and tumor volume measures began. The mice were placed within the magnetic device once or twice a day.

At termination of the study, the primary tumors were dissected from the backs of the mice, weighed, bisected on a plane perpendicular to the backs of the mice and frozen in O.C.T. Compound (Tissue-Tek, Elkhardt, IN). Cryosections, cut approximately 12–15 μM thick, were placed on negatively charged 100 μM gap ChemMate slides (Ventana, Tucson, AZ) air dried for at least 24 hours and fixed in acetone. Immunohistochemical staining was performed on a TechMate Automated Staining System (Ventana) with the ChemMate buffer solutions kit (Ventana). Sections from each tumor were stained with either monoclonal rat anti-mouse CD-31 (PharMingen, San Diego, CA) or a rat IgG2a isotype control (Pharmingen, San Diego, CA to demonstrate specificity) followed by biotinylated rabbit anti-rat immunoglobulin (Dako, Carpinteria, CA). Binding was demonstrated with streptavidin-horseradish peroxidase (Vector Laboratories, Burlingame, CA) and diaminobenzidine (Vector). Slides were dehydrated, coverslipped with Permount (Fisher, Atlanta, GA), and analyzed. CD31 is a 130 kDa integral membrane protein that mediates cell-to-cell adhesion and is expressed on the surface of endothelial cells. The positive staining was identified and quantified using phase contract microscopy and grid intercept point counting.

Morphometric analyses for the percent of necrotic tissue, viable fraction and CD 31 positive was performed on a subset of 51 tumors randomly sampled from each treatment group. Tumor sections stained for CD 31 but lacking a counterstain were studied using phase contrast microscopy. This optical technique allowed differentiation of necrotic, viable and CD 31 stained regions of each tumor. Grid intercept point counting was used to estimate the fraction of an area covered by specific structures. To perform intercept point counting, an ocular grid is placed in the microscope eyepiece to superimpose a grid image over the microscope field. The number of intercepts overlying each structure, divided by the total number of intercepts on the grid estimated the fraction of the area covered by the structure.

Histopathological evaluation of brain, small intestine, kidney, heart, liver and spleen was performed on 5 μM thick sections of formalin fixed, paraffin embedded tissue that were stained with hematoxylin and eosin. Samples were from all mice in the study groups.

Statistical analyses: All data on each tumor were entered into an Excel spreadsheet. Excel was used to generate all calculated variables. SAS or Prism™ software (Prism 5™, Graphpad Software, San Diego, CA) were used to determine means and standard deviations and for analysis of variance (ANOVA) followed by Student-Newman-Keuls or Bonferroni post-hoc tests. Parametric types of analyses can be performed if: 1) the distribution of the data is not significantly different from a normal distribution, 2) the variances of the groups are not significantly different and 3) the treatment groups are independent. Data were found to fit the requirements for parametric analyses.

Tumor volume at each time point, by each treatment was analyzed by 2-way ANOVA followed by a Bonferroni post-hoc test using Prism™.

Prism™ was used to generate all graphs and for analyses of the graphical data. Non-linear regression analysis using an exponential growth fit was used for the growth curves then statistical differences between the curves were calculated using an F-test. The general exponential growth curve is: Y = Y0*exp(k*X) where k is the rate constant (reciprocal of the X axis time units) and X is the time. The doubling time is an estimate of the time for tumor size to double is computed as the ln(2)/K.

A one phase decay model best fit the CD 31 necrotic fraction data. The general formula for this model is: Y = (Y0 - Plateau)*exp(−K*X) + Plateau. Where ‘plateau’ is the point at which the curve levels (i.e. more CD 31 positive did not further decrease the necrotic fraction, 0.03 for this equation) and k is the rate constant (0.12 for this equation/% of CD 31 positive). This makes sense because the necrosis is related to the square of the distance from a blood vessel, if blood vessels are few, the amount of necrosis will be high.

## Abbreviations

ANOVA: Analyses of variance
EMF: Electromagnetic field
SNK: Student-Newman-Keuls posttest
